# Poly[[tetra­aqua­bis­(μ_3_-1*H*-imidazole-4,5-dicarboxyl­ato)tetra­kis­(μ_2_-1*H*-imidazole-4,5-dicarboxyl­ato)tricobalt(II)diytterbium(III)] dihydrate]

**DOI:** 10.1107/S1600536811028285

**Published:** 2011-07-23

**Authors:** Li-Cai Zhu

**Affiliations:** a School of Chemistry and Environment, South China Normal University, Guangzhou 510631, People’s Republic of China

## Abstract

The asymmetric unit of the title compound, {[Co_3_Yb_2_(C_5_H_2_N_2_O_4_)_6_(H_2_O)_4_]·2H_2_O}_*n*_, contains one Yb^III^ ion, two Co^II^ ions (one situated on an inversion centre), three imidazole-4,5-dicarboxyl­ate ligands, two coordinated water mol­ecules and one uncoordinated water mol­ecule. The Yb^III^ ion is seven-coordinated, in a monocapped trigonal prismatic coordination geometry, by six O atoms from three imidazole-4,5-dicarboxyl­ate ligands and one water O atom. Both Co^II^ ions are six-coordinated in a slightly distorted octa­hedral geometry. The Co^II^ ion that is located on an inversion center is coordinated by two O atoms from two water mol­ecules, as well as two O atoms and two N atoms from two imidazole-4,5-dicarboxyl­ate ligands. The second Co^II^ ion is bonded to four O atoms and two N atoms from four imidazole-4,5-dicarboxyl­ate ligands. These metal coordination units are connected by bridging imidazole-4,5-dicarboxyl­ate ligands, generating a three-dimensional network. The crystal structure is further stabilized by N—H⋯O, O—H⋯O and C—H⋯O hydrogen-bonding inter­actions involving the water mol­ecules and the imidazole-4,5-dicarboxyl­ate ligands.

## Related literature

For lanthanide–transition metal heterometallic complexes with bridging multifunctional organic ligands, see: Cheng *et al.* (2006 ▶); Kuang *et al.* (2007 ▶); Sun *et al.* (2006 ▶); Zhu *et al.* (2010 ▶). 
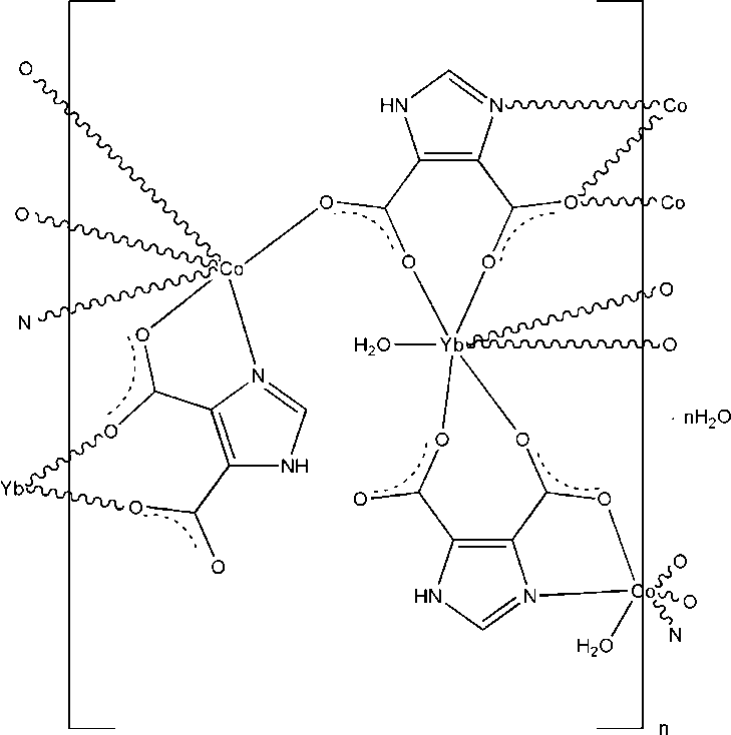


## Experimental

### 

#### Crystal data

[Co_3_Yb_2_(C_5_H_2_N_2_O_4_)_6_(H_2_O)_4_]·2H_2_O
*M* *_r_* = 1555.48
Triclinic, 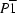

*a* = 7.0413 (4) Å
*b* = 8.3538 (5) Å
*c* = 17.8755 (10) Å
α = 95.546 (1)°
β = 96.886 (1)°
γ = 97.177 (1)°
*V* = 1029.03 (10) Å^3^
*Z* = 1
Mo *K*α radiation
μ = 5.81 mm^−1^
*T* = 296 K
0.20 × 0.18 × 0.15 mm

#### Data collection

Bruker APEXII area-detector diffractometer
Absorption correction: multi-scan (*SADABS*; Sheldrick, 1996 ▶) *T*_min_ = 0.325, *T*_max_ = 0.418
5347 measured reflections
3640 independent reflections
3350 reflections with *I* > 2σ(*I*)
*R*_int_ = 0.019

#### Refinement

*R*[*F* ^2^ > 2σ(*F* ^2^)] = 0.023
*wR*(*F* ^2^) = 0.054
*S* = 1.04
3640 reflections
376 parameters
12 restraints
H atoms treated by a mixture of independent and constrained refinement
Δρ_max_ = 0.78 e Å^−3^
Δρ_min_ = −0.78 e Å^−3^

### 

Data collection: *APEX2* (Bruker, 2007 ▶); cell refinement: *SAINT* (Bruker, 2007 ▶); data reduction: *SAINT*; program(s) used to solve structure: *SHELXS97* (Sheldrick, 2008 ▶); program(s) used to refine structure: *SHELXL97* (Sheldrick, 2008 ▶); molecular graphics: *XP* in *SHELXTL* (Sheldrick, 2008 ▶); software used to prepare material for publication: *SHELXL97*.

## Supplementary Material

Crystal structure: contains datablock(s) I, global. DOI: 10.1107/S1600536811028285/su2289sup1.cif

Structure factors: contains datablock(s) I. DOI: 10.1107/S1600536811028285/su2289Isup2.hkl

Additional supplementary materials:  crystallographic information; 3D view; checkCIF report

## Figures and Tables

**Table 1:** Hydrogen-bond geometry (Å, °)

*D*—H⋯*A*	*D*—H	H⋯*A*	*D*⋯*A*	*D*—H⋯*A*
N2—H1⋯O12^i^	0.87 (3)	2.18 (3)	3.036 (4)	172 (3)
O1*W*—H1*W*⋯O12^ii^	0.82 (4)	1.93 (4)	2.747 (4)	173 (4)
N4—H2⋯O9^iii^	0.86 (3)	2.07 (3)	2.909 (4)	166 (5)
O1*W*—H2*W*⋯O12^iv^	0.80 (3)	2.07 (4)	2.808 (4)	153 (5)
O2*W*—H3*W*⋯O3^v^	0.80 (4)	2.08 (4)	2.870 (5)	168 (5)
N6—H4⋯O1*W*^vi^	0.87 (3)	2.13 (3)	2.948 (5)	157 (4)
N6—H4⋯O3	0.87 (3)	2.53 (4)	3.026 (5)	117 (3)
O2*W*—H4*W*⋯O3*W*	0.81 (3)	1.91 (4)	2.686 (5)	159 (5)
O3*W*—H5*W*⋯O8	0.86 (4)	2.09 (4)	2.928 (4)	165 (5)
O3*W*—H6*W*⋯O2^vi^	0.87 (4)	2.08 (5)	2.904 (4)	158 (4)
C3—H3⋯O2*W*^vii^	0.93	2.43	3.365 (5)	178
C13—H13⋯O4	0.93	2.39	3.198 (5)	145
C13—H13⋯O7	0.93	2.46	3.232 (5)	141

Symmetry codes: (i) 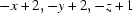; (ii) 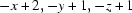; (iii) 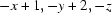; (iv) 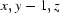; (v) 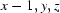; (vi) 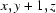; (vii) 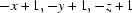.

**Fig. 1.**
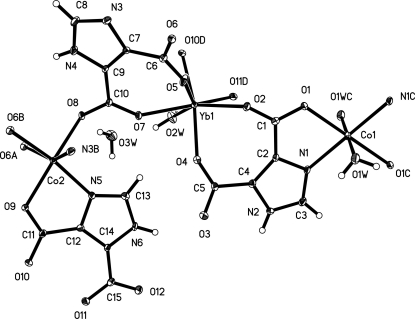
The asymmetric unit of the title compound showing the atomic-numbering scheme and displacement ellipsoids drawn at the 30% probability level. Symmetry codes: (A) x, 1 + y, z; (B) 1 - x, 1 - y, -z; (C) 1 - x, -y, 1 - z; (D) -1 + x, -1 + y, z.

**Fig. 2.**
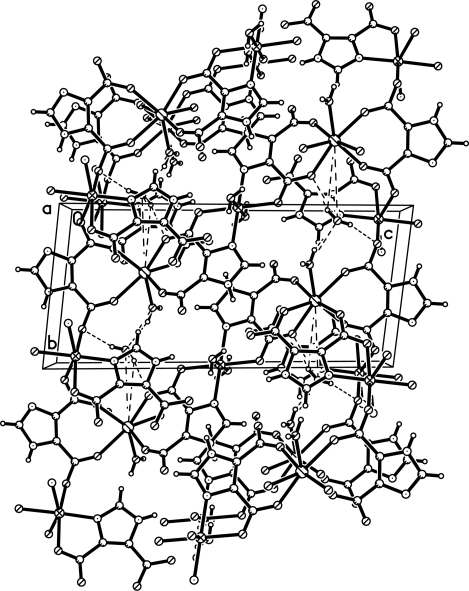
A view of the three-dimensional structure of the title compound. The hydrogen bonding interactions are shown as dashed lines (see Table 1 for details).

## supplementary crystallographic information

### Comment

In the past few years increasing interest has been shown in lanthanide-transition metal heterometallic complexs with bridging multifunctionnal organic ligands, not only because of their impressive topological structures, but also due to their versatile applications in ion exchange, magnetism, bimetallic catalysis and as luminescent probes (Cheng *et al.*, 2006; Kuang *et al.*, 2007; Sun *et al.*, 2006; Zhu *et al.*, 2010). As an extension of this research the structure of the title compound, a new heterometallic coordination polymer, is presented herein.

The asymmetric unit of the title compound (Fig. 1), contains one Yb^III^ ion, one and a half Co^II^ ions, three imidazole-4, 5-dicarboxylate ligands, two coordinated water molecules and one uncoordinated water molecule. The Yb^III^ ion is seven-coordinated in a monocapped trigonal prismatic coordination geometry by six O atoms from three imidazole-4,5-dicarboxylate ligands and one water O atom. Both Co^II^ ions are six-coordinated in a slightly distorted octahedral geometry. The Co1 ion lies on an inversion center and is coordinated with two O atoms from two coordinated water molecules as well as two O atoms and two N atoms from two imidazole-4, 5-dicarboxylate ligands. The Co2 ion is bonded to four O atoms and two N atoms from four imidazole-4,5-dicarboxylate ligands.

These metal coordination units are connected by bridging imidazole-4, 5-dicarboxylate ligands, generating a three-dimensional network (Fig. 2). The crystal structure is further stabilized by N—H···O, O—H···O, and C—H···O hydrogen-bonding interactions involving the water molecules, and the imidazole-4, 5-dicarboxylate ligands (Table 1).

### Experimental

A mixture of CoSO_4_.7H_2_O(0.028 g, 0.1 mmol), Yb_2_O_3_(0.099 g, 0.25 mmol), imidazole-4,5-dicarboxylic acid (0.156 g, 1 mmol), and H_2_O(10 ml) was sealed in a 20 ml Teflon-lined reaction vessel at 443 K for 5 days then slowly cooled to room temperature. The product was collected by filtration, washed with water and air-dried. Red block-like crystals suitable for X-ray analysis were obtained.

### Refinement

The NH and water H-atoms were located in difference Fourier maps and were refined isotropically with distance restraints: N—H = 0.87 (2) Å, O—H = 0.82 (2) or 0.86 (2) Å with *U*_iso_(H) = 1.5 *U*_eq_(N,O). The C-bound H-atoms were positioned geometrically and refined as riding: C—H = 0.93 Å with *U*_iso_(H) = 1.2 *U*_eq_(C).

### Crystal data

**Table:**

[Co_3_Yb_2_(C_5_H_2_N_2_O_4_)_6_(H_2_O)_4_]·2H_2_O	*Z* = 1
*M**_r_* = 1555.48	*F*(000) = 749
Triclinic, *P*1	*D*_x_ = 2.510 Mg m^−^^3^
Hall symbol: -P 1	Mo *K*α radiation, λ = 0.71073 Å
*a* = 7.0413 (4) Å	Cell parameters from 3204 reflections
*b* = 8.3538 (5) Å	θ = 2.5–28.0°
*c* = 17.8755 (10) Å	µ = 5.81 mm^−^^1^
α = 95.546 (1)°	*T* = 296 K
β = 96.886 (1)°	Block, red
γ = 97.177 (1)°	0.20 × 0.18 × 0.15 mm
*V* = 1029.03 (10) Å^3^	

### Data collection

**Table:**

Bruker APEXII area-detector diffractometer	3640 independent reflections
Radiation source: fine-focus sealed tube	3350 reflections with *I* > 2σ(*I*)
graphite	*R*_int_ = 0.019
φ and ω scans	θ_max_ = 25.2°, θ_min_ = 2.3°
Absorption correction: multi-scan (*SADABS*; Sheldrick, 1996)	*h* = −8→8
*T*_min_ = 0.325, *T*_max_ = 0.418	*k* = −9→10
5347 measured reflections	*l* = −19→21

### Refinement

**Table:**

Refinement on *F*^2^	Primary atom site location: structure-invariant direct methods
Least-squares matrix: full	Secondary atom site location: difference Fourier map
*R*[*F*^2^ > 2σ(*F*^2^)] = 0.023	Hydrogen site location: inferred from neighbouring sites
*wR*(*F*^2^) = 0.054	H atoms treated by a mixture of independent and constrained refinement
*S* = 1.04	*w* = 1/[σ^2^(*F*_o_^2^) + (0.025*P*)^2^ + 0.2573*P*] where *P* = (*F*_o_^2^ + 2*F*_c_^2^)/3
3640 reflections	(Δ/σ)_max_ = 0.001
376 parameters	Δρ_max_ = 0.78 e Å^−^^3^
12 restraints	Δρ_min_ = −0.78 e Å^−^^3^

### Special details

**Table:**

Geometry. All e.s.d.'s (except the e.s.d. in the dihedral angle between two l.s. planes) are estimated using the full covariance matrix. The cell e.s.d.'s are taken into account individually in the estimation of e.s.d.'s in distances, angles and torsion angles; correlations between e.s.d.'s in cell parameters are only used when they are defined by crystal symmetry. An approximate (isotropic) treatment of cell e.s.d.'s is used for estimating e.s.d.'s involving l.s. planes.
Refinement. Refinement of *F*^2^ against ALL reflections. The weighted *R*-factor *wR* and goodness of fit *S* are based on *F*^2^, conventional *R*-factors *R* are based on *F*, with *F* set to zero for negative *F*^2^. The threshold expression of *F*^2^ > σ(*F*^2^) is used only for calculating *R*-factors(gt) *etc*. and is not relevant to the choice of reflections for refinement. *R*-factors based on *F*^2^ are statistically about twice as large as those based on *F*, and *R*- factors based on ALL data will be even larger.

### Fractional atomic coordinates and isotropic or equivalent isotropic displacement parameters (Å^2^)

**Table:**

	*x*	*y*	*z*	*U*_iso_*/*U*_eq_	
Yb1	0.29829 (3)	0.41070 (2)	0.243626 (10)	0.01363 (7)	
Co1	0.5000	0.0000	0.5000	0.01581 (18)	
Co2	0.61668 (8)	0.93516 (6)	0.07597 (3)	0.01372 (13)	
C1	0.4567 (6)	0.1646 (5)	0.3642 (2)	0.0139 (9)	
C2	0.5695 (6)	0.2898 (5)	0.4232 (2)	0.0144 (9)	
C3	0.7118 (6)	0.3644 (5)	0.5375 (2)	0.0200 (9)	
H3	0.7531	0.3657	0.5890	0.024*	
C4	0.6687 (6)	0.4428 (5)	0.4231 (2)	0.0149 (9)	
C5	0.7011 (6)	0.5510 (5)	0.3626 (2)	0.0183 (9)	
C6	0.3753 (6)	0.2247 (5)	0.0775 (2)	0.0147 (9)	
C7	0.2950 (6)	0.3342 (5)	0.0250 (2)	0.0131 (8)	
C8	0.1892 (6)	0.3962 (5)	−0.0851 (2)	0.0210 (10)	
H8	0.1429	0.3890	−0.1365	0.025*	
C9	0.2778 (6)	0.4982 (5)	0.0341 (2)	0.0142 (9)	
C10	0.3400 (6)	0.6297 (5)	0.0970 (2)	0.0147 (9)	
C11	0.8955 (6)	1.2012 (5)	0.1572 (2)	0.0155 (9)	
C12	0.8402 (6)	1.1020 (5)	0.2173 (2)	0.0139 (8)	
C13	0.6907 (6)	0.8844 (5)	0.2507 (2)	0.0222 (10)	
H13	0.6135	0.7851	0.2494	0.027*	
C14	0.8922 (6)	1.1069 (5)	0.2941 (2)	0.0132 (8)	
C15	1.0131 (6)	1.2253 (5)	0.3539 (2)	0.0153 (9)	
O1	0.4104 (4)	0.0283 (3)	0.38480 (16)	0.0192 (6)	
O2	0.4150 (4)	0.1990 (3)	0.29715 (16)	0.0213 (7)	
O3	0.8522 (4)	0.6467 (4)	0.37243 (18)	0.0270 (7)	
O4	0.5683 (5)	0.5394 (4)	0.30733 (18)	0.0297 (8)	
O5	0.4029 (5)	0.2590 (3)	0.14763 (16)	0.0240 (7)	
O6	0.4133 (4)	0.0941 (3)	0.04421 (15)	0.0155 (6)	
O7	0.3723 (4)	0.5930 (3)	0.16348 (16)	0.0205 (7)	
O8	0.3603 (4)	0.7716 (3)	0.07878 (15)	0.0185 (6)	
O9	0.8149 (4)	1.1523 (3)	0.09059 (15)	0.0190 (6)	
O10	1.0184 (4)	1.3279 (3)	0.17219 (16)	0.0235 (7)	
O11	1.1107 (4)	1.3474 (3)	0.33394 (16)	0.0205 (7)	
O12	1.0107 (4)	1.2009 (3)	0.42250 (16)	0.0203 (7)	
N1	0.5995 (5)	0.2438 (4)	0.49534 (19)	0.0155 (7)	
N2	0.7592 (5)	0.4862 (4)	0.4960 (2)	0.0175 (8)	
N3	0.2427 (5)	0.2739 (4)	−0.05004 (19)	0.0180 (8)	
N4	0.2106 (5)	0.5326 (4)	−0.0366 (2)	0.0184 (8)	
N5	0.7125 (5)	0.9638 (4)	0.19128 (19)	0.0168 (8)	
N6	0.7947 (5)	0.9664 (4)	0.3127 (2)	0.0195 (8)	
H1	0.824 (6)	0.579 (3)	0.515 (2)	0.029*	
H2	0.190 (7)	0.628 (3)	−0.047 (3)	0.029*	
H4	0.801 (7)	0.937 (5)	0.3580 (15)	0.029*	
O1W	0.7736 (4)	−0.0471 (4)	0.47573 (18)	0.0246 (7)	
H1W	0.830 (6)	−0.099 (5)	0.506 (2)	0.037*	
H2W	0.842 (6)	0.037 (3)	0.475 (3)	0.037*	
O2W	0.1488 (5)	0.6362 (4)	0.2758 (2)	0.0290 (8)	
H3W	0.055 (5)	0.640 (5)	0.297 (3)	0.044*	
H4W	0.181 (7)	0.728 (3)	0.266 (3)	0.044*	
O3W	0.1955 (5)	0.9104 (4)	0.2090 (2)	0.0338 (8)	
H5W	0.250 (7)	0.888 (6)	0.1694 (19)	0.051*	
H6W	0.281 (6)	0.978 (6)	0.240 (2)	0.051*	

### Atomic displacement parameters (Å^2^)

**Table:**

	*U*^11^	*U*^22^	*U*^33^	*U*^12^	*U*^13^	*U*^23^
Yb1	0.01749 (11)	0.01215 (10)	0.00990 (10)	−0.00135 (7)	−0.00088 (7)	0.00222 (7)
Co1	0.0187 (4)	0.0159 (4)	0.0130 (4)	0.0003 (3)	0.0010 (3)	0.0064 (3)
Co2	0.0201 (3)	0.0096 (3)	0.0100 (3)	0.0011 (2)	−0.0023 (2)	0.0004 (2)
C1	0.013 (2)	0.015 (2)	0.014 (2)	0.0057 (17)	0.0015 (17)	−0.0011 (17)
C2	0.017 (2)	0.014 (2)	0.012 (2)	0.0024 (17)	0.0014 (17)	0.0046 (17)
C3	0.027 (3)	0.020 (2)	0.012 (2)	0.0045 (19)	−0.0026 (18)	0.0021 (18)
C4	0.012 (2)	0.017 (2)	0.016 (2)	0.0047 (17)	0.0015 (17)	0.0040 (17)
C5	0.023 (2)	0.013 (2)	0.019 (2)	0.0018 (18)	−0.0009 (19)	0.0052 (18)
C6	0.014 (2)	0.017 (2)	0.013 (2)	0.0027 (17)	0.0016 (17)	0.0026 (17)
C7	0.015 (2)	0.013 (2)	0.010 (2)	0.0000 (17)	0.0013 (16)	−0.0002 (16)
C8	0.029 (3)	0.023 (2)	0.011 (2)	0.007 (2)	−0.0036 (18)	0.0017 (18)
C9	0.018 (2)	0.015 (2)	0.009 (2)	0.0014 (17)	−0.0010 (17)	0.0022 (16)
C10	0.014 (2)	0.019 (2)	0.013 (2)	0.0029 (17)	0.0020 (16)	0.0061 (17)
C11	0.017 (2)	0.016 (2)	0.014 (2)	0.0041 (18)	−0.0013 (17)	0.0034 (17)
C12	0.014 (2)	0.011 (2)	0.016 (2)	0.0025 (16)	−0.0012 (17)	0.0008 (17)
C13	0.025 (3)	0.019 (2)	0.019 (2)	−0.0059 (19)	−0.0009 (19)	0.0025 (19)
C14	0.014 (2)	0.014 (2)	0.012 (2)	0.0029 (17)	−0.0001 (16)	0.0033 (17)
C15	0.015 (2)	0.017 (2)	0.015 (2)	0.0079 (18)	0.0012 (17)	0.0022 (18)
O1	0.0273 (17)	0.0132 (15)	0.0150 (16)	−0.0028 (13)	−0.0022 (13)	0.0040 (12)
O2	0.0328 (18)	0.0164 (15)	0.0131 (16)	0.0015 (13)	−0.0028 (13)	0.0036 (12)
O3	0.0205 (17)	0.0268 (17)	0.0309 (19)	−0.0070 (14)	−0.0046 (14)	0.0122 (15)
O4	0.0334 (19)	0.0232 (17)	0.0273 (18)	−0.0096 (15)	−0.0108 (15)	0.0125 (15)
O5	0.042 (2)	0.0211 (16)	0.0106 (16)	0.0132 (15)	0.0020 (14)	0.0009 (13)
O6	0.0248 (16)	0.0087 (14)	0.0128 (15)	0.0046 (12)	0.0008 (12)	−0.0012 (12)
O7	0.0355 (19)	0.0133 (14)	0.0113 (15)	−0.0018 (13)	0.0013 (13)	0.0026 (12)
O8	0.0272 (17)	0.0119 (14)	0.0156 (15)	−0.0011 (13)	0.0001 (13)	0.0059 (12)
O9	0.0251 (17)	0.0167 (15)	0.0115 (15)	−0.0068 (13)	−0.0031 (13)	0.0029 (12)
O10	0.0255 (17)	0.0207 (16)	0.0194 (17)	−0.0112 (14)	−0.0052 (13)	0.0063 (13)
O11	0.0247 (17)	0.0195 (16)	0.0146 (16)	−0.0056 (13)	0.0011 (13)	0.0006 (13)
O12	0.0239 (17)	0.0230 (16)	0.0138 (16)	0.0018 (13)	0.0027 (13)	0.0028 (13)
N1	0.0207 (19)	0.0152 (18)	0.0110 (18)	0.0034 (15)	0.0008 (14)	0.0039 (14)
N2	0.021 (2)	0.0134 (18)	0.0153 (19)	−0.0031 (15)	−0.0032 (15)	0.0006 (15)
N3	0.023 (2)	0.0166 (18)	0.0128 (18)	0.0017 (15)	−0.0025 (15)	−0.0007 (15)
N4	0.027 (2)	0.0142 (18)	0.0155 (19)	0.0071 (16)	0.0000 (16)	0.0045 (15)
N5	0.023 (2)	0.0114 (17)	0.0144 (18)	−0.0020 (15)	−0.0007 (15)	0.0023 (14)
N6	0.028 (2)	0.0189 (19)	0.0108 (19)	−0.0012 (16)	−0.0019 (16)	0.0080 (16)
O1W	0.0204 (18)	0.0289 (18)	0.0264 (18)	0.0038 (14)	0.0031 (14)	0.0127 (15)
O2W	0.036 (2)	0.0202 (17)	0.035 (2)	0.0044 (16)	0.0169 (16)	0.0079 (15)
O3W	0.041 (2)	0.0250 (19)	0.035 (2)	0.0032 (16)	0.0108 (17)	−0.0038 (16)

### Geometric parameters (Å, °)

**Table:**

Yb1—O4	2.191 (3)	C7—C9	1.386 (5)
Yb1—O10^i^	2.209 (3)	C8—N3	1.320 (5)
Yb1—O7	2.246 (3)	C8—N4	1.344 (5)
Yb1—O11^i^	2.266 (3)	C8—H8	0.9300
Yb1—O5	2.282 (3)	C9—N4	1.366 (5)
Yb1—O2	2.285 (3)	C9—C10	1.479 (6)
Yb1—O2W	2.328 (3)	C10—O8	1.254 (5)
Co1—N1^ii^	2.082 (3)	C10—O7	1.257 (5)
Co1—N1	2.082 (3)	C11—O9	1.262 (5)
Co1—O1W	2.100 (3)	C11—O10	1.265 (5)
Co1—O1W^ii^	2.100 (3)	C11—C12	1.478 (5)
Co1—O1^ii^	2.125 (3)	C12—C14	1.374 (5)
Co1—O1	2.125 (3)	C12—N5	1.376 (5)
Co2—N5	2.072 (3)	C13—N5	1.320 (5)
Co2—O9	2.120 (3)	C13—N6	1.330 (6)
Co2—O6^iii^	2.120 (3)	C13—H13	0.9300
Co2—O8	2.132 (3)	C14—N6	1.374 (5)
Co2—O6^iv^	2.136 (3)	C14—C15	1.487 (6)
Co2—N3^iii^	2.152 (3)	C15—O11	1.262 (5)
C1—O1	1.247 (5)	C15—O12	1.264 (5)
C1—O2	1.268 (5)	O6—Co2^iii^	2.120 (3)
C1—C2	1.489 (5)	O6—Co2^v^	2.136 (3)
C2—C4	1.378 (6)	O10—Yb1^vi^	2.209 (3)
C2—N1	1.380 (5)	O11—Yb1^vi^	2.266 (3)
C3—N1	1.312 (5)	N2—H1	0.867 (19)
C3—N2	1.347 (5)	N3—Co2^iii^	2.152 (3)
C3—H3	0.9300	N4—H2	0.863 (19)
C4—N2	1.374 (5)	N6—H4	0.865 (19)
C4—C5	1.496 (5)	O1W—H1W	0.82 (4)
C5—O3	1.232 (5)	O1W—H2W	0.804 (19)
C5—O4	1.264 (5)	O2W—H3W	0.80 (4)
C6—O5	1.245 (5)	O2W—H4W	0.811 (19)
C6—O6	1.265 (5)	O3W—H5W	0.86 (4)
C6—C7	1.484 (5)	O3W—H6W	0.87 (4)
C7—N3	1.376 (5)		
			
O4—Yb1—O10^i^	168.77 (11)	O5—C6—C7	122.9 (4)
O4—Yb1—O7	80.61 (11)	O6—C6—C7	113.7 (3)
O10^i^—Yb1—O7	89.98 (10)	N3—C7—C9	109.4 (3)
O4—Yb1—O11^i^	104.42 (11)	N3—C7—C6	117.8 (3)
O10^i^—Yb1—O11^i^	79.80 (11)	C9—C7—C6	132.4 (4)
O7—Yb1—O11^i^	144.82 (11)	N3—C8—N4	110.9 (4)
O4—Yb1—O5	102.77 (12)	N3—C8—H8	124.6
O10^i^—Yb1—O5	80.83 (11)	N4—C8—H8	124.6
O7—Yb1—O5	76.85 (10)	N4—C9—C7	104.8 (3)
O11^i^—Yb1—O5	133.18 (10)	N4—C9—C10	120.7 (3)
O4—Yb1—O2	80.51 (11)	C7—C9—C10	133.8 (4)
O10^i^—Yb1—O2	110.72 (10)	O8—C10—O7	124.9 (4)
O7—Yb1—O2	140.86 (11)	O8—C10—C9	116.2 (3)
O11^i^—Yb1—O2	73.46 (11)	O7—C10—C9	118.9 (3)
O5—Yb1—O2	74.36 (10)	O9—C11—O10	122.2 (4)
O4—Yb1—O2W	88.67 (13)	O9—C11—C12	116.5 (4)
O10^i^—Yb1—O2W	82.67 (12)	O10—C11—C12	121.3 (4)
O7—Yb1—O2W	72.93 (11)	C14—C12—N5	109.5 (3)
O11^i^—Yb1—O2W	72.42 (11)	C14—C12—C11	136.1 (4)
O5—Yb1—O2W	145.39 (11)	N5—C12—C11	114.2 (3)
O2—Yb1—O2W	140.19 (11)	N5—C13—N6	110.4 (4)
N1^ii^—Co1—N1	180.000 (1)	N5—C13—H13	124.8
N1^ii^—Co1—O1W	93.48 (13)	N6—C13—H13	124.8
N1—Co1—O1W	86.52 (13)	N6—C14—C12	104.4 (3)
N1^ii^—Co1—O1W^ii^	86.52 (13)	N6—C14—C15	120.5 (3)
N1—Co1—O1W^ii^	93.48 (13)	C12—C14—C15	135.1 (4)
O1W—Co1—O1W^ii^	180.000 (1)	O11—C15—O12	122.9 (4)
N1^ii^—Co1—O1^ii^	77.93 (12)	O11—C15—C14	118.5 (4)
N1—Co1—O1^ii^	102.07 (12)	O12—C15—C14	118.6 (4)
O1W—Co1—O1^ii^	88.35 (12)	C1—O1—Co1	116.7 (3)
O1W^ii^—Co1—O1^ii^	91.65 (12)	C1—O2—Yb1	135.6 (3)
N1^ii^—Co1—O1	102.07 (12)	C5—O4—Yb1	149.7 (3)
N1—Co1—O1	77.93 (12)	C6—O5—Yb1	141.9 (3)
O1W—Co1—O1	91.65 (12)	C6—O6—Co2^iii^	118.5 (2)
O1W^ii^—Co1—O1	88.35 (12)	C6—O6—Co2^v^	131.8 (3)
O1^ii^—Co1—O1	180.0	Co2^iii^—O6—Co2^v^	103.63 (11)
N5—Co2—O9	77.03 (12)	C10—O7—Yb1	145.5 (3)
N5—Co2—O6^iii^	166.83 (12)	C10—O8—Co2	130.1 (3)
O9—Co2—O6^iii^	95.87 (10)	C11—O9—Co2	116.9 (3)
N5—Co2—O8	97.27 (12)	C11—O10—Yb1^vi^	138.7 (3)
O9—Co2—O8	160.59 (11)	C15—O11—Yb1^vi^	139.5 (3)
O6^iii^—Co2—O8	92.93 (11)	C3—N1—C2	106.8 (3)
N5—Co2—O6^iv^	113.16 (12)	C3—N1—Co1	138.9 (3)
O9—Co2—O6^iv^	82.90 (11)	C2—N1—Co1	113.1 (3)
O6^iii^—Co2—O6^iv^	76.37 (11)	C3—N2—C4	108.2 (3)
O8—Co2—O6^iv^	82.41 (11)	C3—N2—H1	124 (3)
N5—Co2—N3^iii^	95.60 (13)	C4—N2—H1	127 (3)
O9—Co2—N3^iii^	111.27 (12)	C8—N3—C7	106.1 (3)
O6^iii^—Co2—N3^iii^	76.47 (11)	C8—N3—Co2^iii^	137.1 (3)
O8—Co2—N3^iii^	87.60 (12)	C7—N3—Co2^iii^	109.9 (3)
O6^iv^—Co2—N3^iii^	150.46 (12)	C8—N4—C9	108.7 (3)
O1—C1—O2	123.8 (4)	C8—N4—H2	127 (3)
O1—C1—C2	116.2 (3)	C9—N4—H2	124 (3)
O2—C1—C2	120.0 (3)	C13—N5—C12	106.3 (3)
C4—C2—N1	108.8 (4)	C13—N5—Co2	138.5 (3)
C4—C2—C1	135.1 (4)	C12—N5—Co2	115.2 (3)
N1—C2—C1	115.9 (3)	C13—N6—C14	109.4 (4)
N1—C3—N2	110.7 (4)	C13—N6—H4	126 (3)
N1—C3—H3	124.6	C14—N6—H4	125 (3)
N2—C3—H3	124.6	Co1—O1W—H1W	115 (4)
N2—C4—C2	105.4 (4)	Co1—O1W—H2W	110 (4)
N2—C4—C5	120.7 (4)	H1W—O1W—H2W	107 (3)
C2—C4—C5	133.8 (4)	Yb1—O2W—H3W	128 (3)
O3—C5—O4	126.0 (4)	Yb1—O2W—H4W	126 (3)
O3—C5—C4	116.9 (4)	H3W—O2W—H4W	106 (3)
O4—C5—C4	117.0 (4)	H5W—O3W—H6W	106 (3)
O5—C6—O6	123.4 (4)		

Symmetry codes: (i) *x*−1, *y*−1, *z*; (ii) −*x*+1, −*y*, −*z*+1; (iii) −*x*+1, −*y*+1, −*z*; (iv) *x*, *y*+1, *z*; (v) *x*, *y*−1, *z*; (vi) *x*+1, *y*+1, *z*.

### Hydrogen-bond geometry (Å, °)

**Table:**

*D*—H···*A*	*D*—H	H···*A*	*D*···*A*	*D*—H···*A*
N2—H1···O12^vii^	0.87 (3)	2.18 (3)	3.036 (4)	172 (3)
O1W—H1W···O12^viii^	0.82 (4)	1.93 (4)	2.747 (4)	173 (4)
N4—H2···O9^ix^	0.86 (3)	2.07 (3)	2.909 (4)	166 (5)
O1W—H2W···O12^v^	0.80 (3)	2.07 (4)	2.808 (4)	153 (5)
O2W—H3W···O3^x^	0.80 (4)	2.08 (4)	2.870 (5)	168 (5)
N6—H4···O1W^iv^	0.87 (3)	2.13 (3)	2.948 (5)	157 (4)
N6—H4···O3	0.87 (3)	2.53 (4)	3.026 (5)	117 (3)
O2W—H4W···O3W	0.81 (3)	1.91 (4)	2.686 (5)	159 (5)
O3W—H5W···O8	0.86 (4)	2.09 (4)	2.928 (4)	165 (5)
O3W—H6W···O2^iv^	0.87 (4)	2.08 (5)	2.904 (4)	158 (4)
C3—H3···O2W^xi^	0.93	2.43	3.365 (5)	178
C13—H13···O4	0.93	2.39	3.198 (5)	145
C13—H13···O7	0.93	2.46	3.232 (5)	141

Symmetry codes: (vii) −*x*+2, −*y*+2, −*z*+1; (viii) −*x*+2, −*y*+1, −*z*+1; (ix) −*x*+1, −*y*+2, −*z*; (v) *x*, *y*−1, *z*; (x) *x*−1, *y*, *z*; (iv) *x*, *y*+1, *z*; (xi) −*x*+1, −*y*+1, −*z*+1.
